# Supplementary material for: Prevalence of vertical movement asymmetries at trot in Standardbred and Swedish Warmblood foals
Source: PLoS One. 2023 Apr 6;18(4):e0284105. doi: 10.1371/journal.pone.0284105 (PMC10079035; doi:10.1371/journal.pone.0284105)
Supplement: Attachment — Submitted filename: Response to Reviewers.docx [file pone.0284105.s003.docx]

Response to Reviewers PONE-D-22-33503

Lines referred to the file “Revised Manuscript with Track Changes”

**Reviewer #1**

Thank you for taking the time to review our manuscript.

This study adequately describes the vertical symmetry of head and pelvis at the trot in young foals. The methods are well described and results are presented in sufficient detail. Please change the name of the IMU system to 'Equinoxes Q with Lameness Locator software (version...). The discussion is broad but generally acceptable.

**Answer:** It has now been corrected to “(Equinosis Q with Lameness Locator, software v1.2r, )” in the data collection section.

**Reviewer #2**

Thank you for all the valuable suggestions and feedback to improve our manuscript.

This paper describes the measurement of gait asymmetries in young foals. This is a potentially important study because there is increasing recognition that measurable asymmetry does not necessarily equate with clinically significant pain-related lameness. However, the study has some limitations, not all of which have been discussed adequately. Ideally these foals should have been assessed on more than one occasion to determine the repeatability of the results. More use could have been made of the video recordings to assess movement and to discuss the factors that may have influenced the results. Young foals are generally not trained to trot in straight lines, with a straight neck and trunk, and at a constant speed - how might this have influenced the results? I think that the only conclusion that can be made is that there was a high prevalence of measurable asymmetry in these young foals; we have no idea whether similar findings would be made on a day-to-day basis, how they may be influenced by early training, nor how these results would change over the ensuing months. It is an over-interpretation in my opinion to suggest that this asymmetry reflects innate congenital alterations in their motion pattern (laterality). There are other factors which need to be considered.

Answer: We agree that the study has some limitations and we have made changes in the discussion to address them, see lines 326-329. “Additionally it would have been of interest to follow the foals for a longer period, to assess the repeatability of results. Repeated measurements could also provide information about inter-individual fluctuations over time, in relation to e.g. handling, training or possible development of pathologies”. It would have been of interest to, as you describe, perform a longitudinal study to assess the repeatability of our results and follow the foals over a longer period. However, due to practical reasons, this was not possible. This study was conducted as a first attempt to gain an understanding about the prevalence of asymmetries in foals, since this has not been looked at previously. Thanks to this study, we have gained some knowledge from which one can generate new questions to investigate the issue further. It was not possible to make this first study large enough to address all potential influencing factors.

All the included sequences, which the results are based on, are from straight line trot. Parts where the foals were not trotting in a straight line or making movements interfering with the aimed measurement (e.g. biting after the mare, violent head movements) were all excluded. The speed of the trot was not controlled as it is described in Starke S. et al. 2013 *“The effect of trotting speed on the evaluation of subtle lameness in horses”*, that objective measurements are not influenced by alternating speeds.

One of our intentions with this manuscript is to highlight that this is a complex subject and that the presence of asymmetries in horses is likely to be influenced by multiple different factors. Throughout the manuscript, different factors such as age, subclinical lameness, breed etc. are suggested

The authors are also perpetuating the 'myth' that an 'owner sound horse' (dreadful term) is potentially a non-lame horse and fail to take into account other factors that could be considered in the differentiation of lame versus non-lame horses.

**Answer:** We realise that this expression can be interpreted in different ways, and have now removed it. Looking at horses perceived as non-lame by their owner is according to us of relevance since we need to address the potential issue of horses training and competing when they are in pain. A majority of sport horses are only assessed by a veterinarian in regard to lameness when the owner perceives or suspects such an issue. By studying horses perceived as non-lame we hope to gain a better understanding about these issues, and thereby contribute to an improved welfare in horses.

Key words are additional words for a search engine and should not be words in the title

**Answer:** Thank you for pointing this out. The keywords have now been changed.

Line 15 Odd way to start the Abstract. I suggest that the first sentence is rewritten.

**Answer:** The sentence has now been rewritten.

Lines 49-51 References needed
Line 53 Reference needed

**Answer:** The references have now been added.

Line 59 what is meant by indicated in this context?

Answer: changed to “observed”

Lines 73-5 Rephrase to make the meaning clearer e.g. Movement asymmetry may to some extent be inherent, although may be influenced by .....

**Answer:** Some changes have been done but we would like to keep its structure as it is.

Line 76 I would use a different word than hypothesise because your study design did not permit testing this hypothesis - unless you set a threshold for prevalence - or compared other studies statistically. You infer in the Discussion that you did test this hypothesis but in reality you did not do so in a robust way. It is nonetheless obvious that there was a high prevalence of asymmetry!

**Answer:** we have now replaced the word in the manuscript.

Line 101 Source of the double adhesive tape?

**Answer:** we have added the source, “(Tesa)”

Lines 104-5 'using tape under the halter lateral of the mane or a headband.' It is not clear what this means

**Answer:** Yes we struggled with this sentence. It is now changed, se lines 104 -107“For many of the foals, the halter had to be secured in place, to prevent it from slipping back on the neck and thereby displaced from the poll. Securing the placement was done with the use of a headband or double adhesive tape placed under the halter on both sides of the mane.” We hope that it is more clear now.

Line 55 painful conditions .... are
Line 64 a horse's (not the)

Line 87 of age

Line 99 dorsal aspect of the pastern

Line 213 asymmetry ... was
Line 218 The aim ... was

Line 262 Thoroughbreds

Line 292 'track their horses' movements'

Line 303 an adult horse

**Answer:** Thank you, all of this has now been changed.

Line 108 How were the foals trotted? Were they free or led? Were they following their dam? This needs to be in M&M, not Results.

**Answer:** This has now been added in M&M, se lines 109-111. “Foals were encouraged to trot by following the mare, who was trotted by hand in a straight line back and forth while the foal was either loose or led close to the mare by a second handler.”

What prior handling had the foals received and was this consistent among the groups?
Had the foals received any prior training to trot in a straight line? How might this have influenced your results?

**Answer**: No foals received any specific prior training in relation to the study. Many of the foals, especially the ones located at the stud farm were planned to be/had already been participating in shows. Whether the owner had trained the foals to trot after the mare is not known. In regard to achieving a situation similar to if a random foal was to be measured, no prior training specific to this study was wanted. Training might also influence the results, which would make generalisations to other populations of foals inappropriate.

“No specific training of the foals was performed prior to the measurements.” Has been added to the data collection section to clarify this, see line 118.

How were the foals encouraged to trot?

**Answer:** Most of the foals followed the mare when she trotted away, while some were encouraged by the researchers gently using their voices.

It is a shame that the foals were not examined on >1 occasion to determine the repeatability of the observations!

**Answer:** Yes this would have been valuable but for practical reasons, it was not possible in the present study.

Line 113 Where were the cameras located - from the side, behind or in front?

**Answer:** We have now added this information, see lines 115-119. “During all measurements the foals were recorded either from the side by a researcher with a hand-held camera, or by fence mounted cameras capturing simultaneously from the side, from behind and from the front.”

Line 199 Table and figure legends should be comprehensive and should be able to be read independently of the text.

Table 2 What is meant by ipsilateral asymmetry, contralateral asymmetry, three limb asymmetry? These terms need to be defined for clarity – not leaving a reader to guess, perhaps incorrectly

**Answer:** Some more descriptions have been added. See lines 205-206, 215-217.

Why was there more right asymmetry for PD min? – 12/13 – needs discussion

**Answer**: We have added sentences to the discussion, addressing this skewed distribution, see lines 286-289. “The foals trotted to similar degree on both sides of the mare and the measurements were performed in both directions to reduce influence of environmental factors (e.g. fences). A preferred side might be discussed but further, more directed, research is needed to draw any conclusions about this skewed distribution.”

How do you interpret the observations related to bilateral forelimb an hindlimb asymmetry, defined as ‘bilateral forelimb asymmetry refers to cases where HDmin and HDmax are attributed to different limbs, and bilateral hindlimb asymmetry to cases where PDmin and PDmax are attributed to different limbs.’ This needs discussion.

**Answer**: Since the stride is separated in a loading and a push of sequence in the analysis, a horse can for example have an asymmetry correlated to the loading phase for one forelimb and an asymmetry correlated to the push of in the other forelimb at the same time.

Why were horses with a lameness grade of 1/5 included in the study? How many foals had grade 1/5 lameness?

**Answer:** We recognize the issue. Since we wanted to look at asymmetries we need to accept some degree of deviation from visually perfect symmetry. We do not know if all asymmetries should be seen as lameness and did not want to exclude this very mild subjectively graded lameness, which might not be due to pain, because then we might have missed important information.

Line 220 What is meant by 'a reaction' in this context?

**Answer:** behaviours such as withdrawal, jumping, biting etc. that you might not expect from an adult horse during clinical examination.

Line 241 From the video recordings can you say what proportion of foals trotted straight?

**Answer:** The sequences we included was of straight line trot. This was ensured using the videos. Most of the foals followed the mare and as long as she was trotted in a straight line, they followed her. Sequences where this was not the case are excluded.

Line 248 What breed of foals? How many? Examined under what circumstances?

**Answer:** 9 Warmblood foals were included, video recordings followed by analysis. We have added to the sentence in the manuscript, see line 257.

Line 255 - 6 'Subjective comparison of prevalence among our foals and older horses revealed higher prevalence of asymmetries among the older SWB horses' - what study does this refer to?
It is rather confusing to have a sentence combining observations in Warmbloods & Standardbreds.

**Answer**: The reference has been added and the sentences has been rewritten. “Subjective comparison of prevalence among our foals and older horses revealed higher prevalence of asymmetries among the older Warmblood horses [1] compared to the SWB foals, potentially reflecting an increase in asymmetries in horses during training. Significant increase in asymmetries has been reported during intense training of trotters, with elevated values persisting for almost three years”

Line 259 Increased with respect to what?

**Answer**: The sentence has been rewritten to make the meaning clearer, see lines 269-270. (in comparison to not training of the horse). “This reflects the supposed outcome based on the presumption of training resulting in an increased risk of repetitive strain injuries and higher age increasing the accumulated risk of traumatic injuries.”

Ref 10 = polo ponies - were these all Thoroughbreds?? - NO
32 Thoroughbreds, 19 Argentinian polo horses and 9 horses of unknown or other breeds

**Answer**: The reference has been removed.

Line 271 ‘Manifestation of a preferred left or right side, referred to as idiosyncratic motor laterality, has been seen among adult horses’ 24. Murphy J, Sutherland A, Arkins S. Idiosyncratic motor laterality in the horse. Appl Anim Behav Sci. 2005;91: 297–310. doi:10.1016/j.applanim.2004.11.001
Please make it clearer what type of laterality you are referring to in this context. ‘Motor’ is not enough. The laterality described in Murphy et al. does not necessarily relate to movement asymmetry in the context of the current paper. There is a real danger that the terms laterality and handedness get confused, used interchangeably and applied to different circumstances, which creates muddle and confusion. It would be helpful if the current authors clearly define what they are inferring by laterality.

**Answer**: We agree that this term should be used with care, and more research is needed. The sentences have been rewritten, see lines 288-283.

Line 277 Statement needs a reference

**Answer:** The meaning of the sentence has been corrected. “Early detection of signs of lameness is essential for an early treatment and thereby a potentially more favourable prognosis.”

Line 281 the term 'owner-sound horses' is vague, non-scientific and inappropriate term which is sadly becoming prevalent in objective gait analysis literature and is potentially misleading.
A better description might be ‘horses perceived by their owners to be non-lame’ - which does not necessarily equate with non-lame!

**Answer**: The term has been replaced to avoid this confusion.

Anyone who has done extensive lameness and performance work with polo ponies, riding horses and trotters will attest to the fact that a high proportion are lame, despite being in regular work. By far the most common reason for a horse not being recommended for purchase at a pre-purchase examination is because of lameness.

**Answer:** We agree.

Line 287 This is a potentially spurious & misleading argument because meloxicam is not a particularly effective drug in improving orthopaedic pain.

**Answer:** Yes this sentence needed clarification. It is now rewritten. “In an attempt to confirm pain as a cause of asymmetries among adult horses, one study treated riding horses in training showing asymmetries with meloxicam (a non-steroidal anti-inflammatory drug with indication for treatment of acute and chronic locomotive disorders) and found that the treatment did not significantly affect asymmetries. These results indicate pain non-responsive to meloxicam or a non-painful cause for the asymmetries [43].”

Line 288 ‘it is difficult to decide how older populations of “owner-sound” horses with asymmetries should be evaluated’
See if asymmetries change under different circumstances e.g., in hand, after flexion tests, on the lunge on both soft and firm surfaces and ridden.
Look at behaviour!
Assess response to diagnostic anaesthesia

**Answer:** We see that the sentence was easily misunderstood, it should have been clearer what we referred to. The sentence has been rewritten since we were not referring to a clinical setting as we understand that you interpreted it as, we meant in relation to the objective measurements done with no lameness evaluation. Hope this is clearer now, see lines 306-310. “If horses are born with vertical asymmetries of the head and pelvis that are unrelated to pain, it is difficult to decide how the older population of horses, perceived as non-lame by owners, but presenting with asymmetries should be evaluated. This in the context of objective gait analysis, with no specific suspicion of lameness. Taking the above into consideration, the individual horse might well serve as its own reference for evaluating objective movement measurements when not related to suspicion of lameness. The availability of smartphones and ongoing rapid technological advances may soon m…”

Line 294 You did not define in your M&M what populations you were comparing with, nor did you test this statistically

**Answer:** You are correct, this is not meant to be in the sentence. It has now been corrected.

Line 297 I unquestionably agree that the use of thresholds is potentially misleading but their use in so-called ‘owner- sound horses’ is potentially misleading as an argument for their lack of validity. It has been demonstrated that a large proportion of horses presumed by their owners to be working comfortably were lame when evaluated by skilled clinicians and had high RHpE scores, supporting the inference that these were genuine pain-induced gait asymmetries.

Line 299 Measurable pelvic asymmetry was also seen in horses assessed by experienced clinicians and judged to be non-lame in hand, on the lunge and ridden
Greve, L. and Dyson, S. (2016) Body lean angle in sound dressage horses in-hand, on the lunge and ridden. Vet. J. 217, 52-57.
They documented measurable pelvic movement asymmetry outside published data in non-lame horses (Warmbloods) assessed by experts

**Answer:** We agree that the origin of asymmetries should be looked into further. However, investigating the causes of asymmetries further falls outside of the scope of this study.

Line 312 Please explain what you mean by lateralisation tests and provide a reference. Please also explain what you mean by 'possible pain evaluations'.

**Answer:** For example limb preference tests, *Murphy J, Sutherland A, Arkins S. Idiosyncratic motor laterality in the horse,* this reference has now been added. Regarding the pain we mean for example diagnostic anaesthesia (the “possible” is removed).

Line 313 The study could have been improved by:
Determining repeatability of results on different occasions
Longitudinal monitoring from young foals and at intervals thereafter

**Answer:** Yes we agree, and further research is needed!
